# Rap1 activity and localization is regulated by Rab40/CRL5 facilitated mono-ubiquitylation.

**DOI:** 10.17912/micropub.biology.001629

**Published:** 2025-06-09

**Authors:** Andrew Neumann, Revathi Sampath, Ke-Jun Han, Rytis Prekeris

**Affiliations:** 1 Cell and Developmental Biology, University of Colorado Anschutz Medical Campus, Aurora, Colorado, United States

## Abstract

The Rap family of GTPases are emerging as major regulators of actin dynamics and cell migration. However, how Rap GTPases are activated and targeted to their subcellular localization remains to be fully understood. Recent work has shown that Rab40/CRL5-dependent mono-ubiquitylation is required for Rap2 activation. Here, we show that Rap1 is also mono-ubiquitylated by a Rab40/CRL5 E3 ubiquitin ligase complex and that Rap1 mono-ubiquitylation is necessary for Rap1 localization to both the plasma membrane and nuclear envelope. In summary, this work shows that Rab40/CRL5 is a major regulator of the activity and spatiotemporal dynamics of the Rap family of GTPases.

**Figure 1. Rab40/CRL5 dependent mono-ubiquitylation regulates Rap1b activation and localization.**
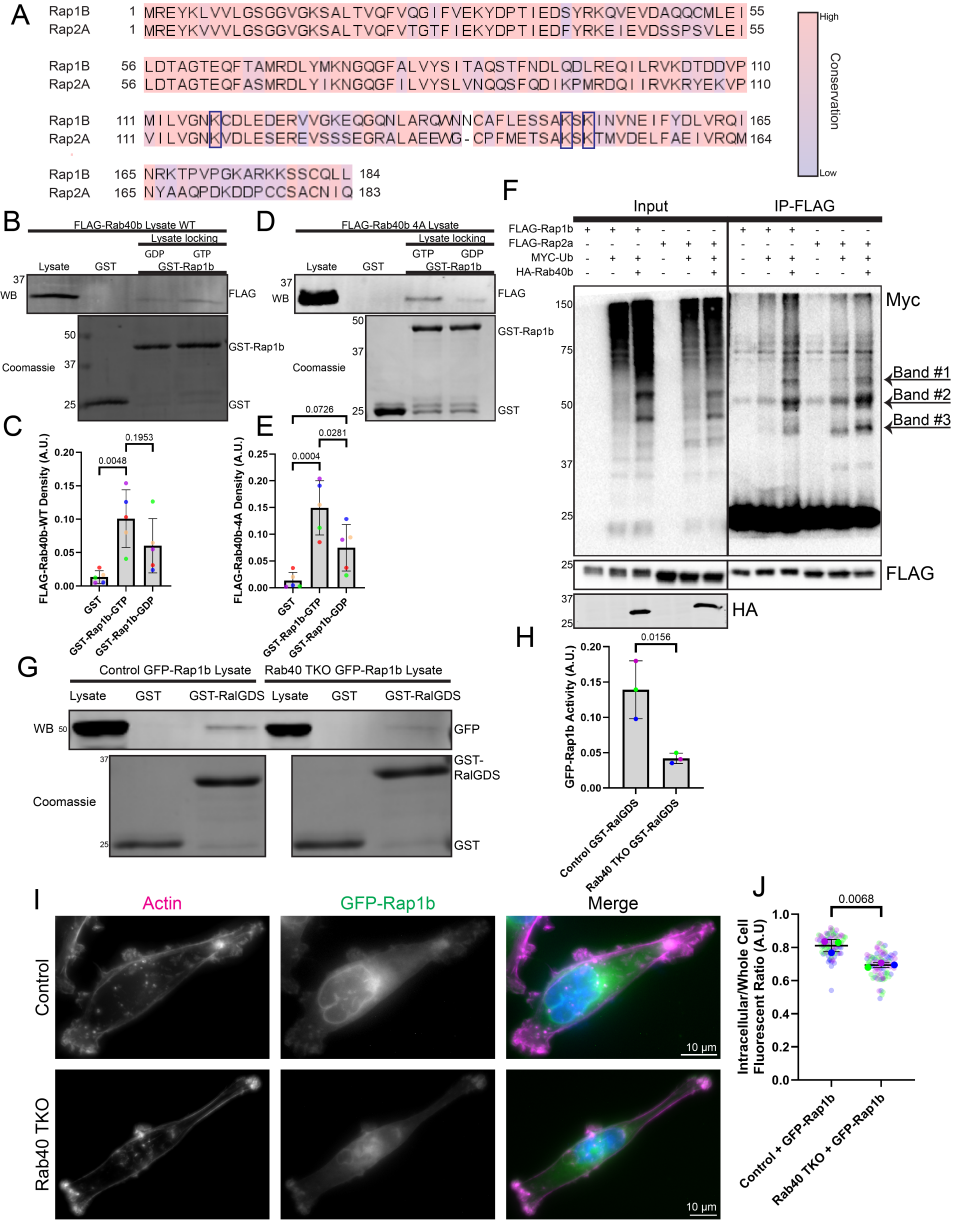
**(A)** alignment of Rap1b and Rap2a made using t-Coffee which demonstrates the high conservation (colored in Jalview by percent identity) between the two proteins and the conservation of Rab40/CRL5 lysine targets (black box). **(B-E)** Lysates from MDA-MB-231 cells overexpressing FLAG-Rab40b-WT or FLAG-Rab40b-4A were incubated with GST-Rap1b beads to assess binding. Panel **(B)** shows a representative western blot of FLAG-Rab40b-WT binding to Rap1b which is quantified in **(C).** Panel **(D)** shows a representative western blot of FLAG-Rab40b-4A binding to Rap1b which is quantified in **(D).** **(F)** Rap1/2 ubiquitylation assay from HEK293T cells. FLAG-Rap1b/Rap2a, MYC-Ub, and HA-Rab40b were transfected into 293T cells. FLAG-Rap1b/Rap2a was immunoprecipitated and MYC staining was used to assess the pattern of Rap1b/Rap2a ubiquitylation. **(G-H)** GST-RalGDS pulldowns were used to assess the activity of GFP-Rap1b in control and Rab40-TKO cells. GST-RalGDS binding to GFP-Rap1b was assessed by western blot and the presence of GST-RalGDS in cell lysates was confirmed by Coomassie blue staining. Quantification of GFP-Rap1b binding to GST-RalGDS normalized to GFP-Rap1b levels in the lysate **(H).** **(I-J)** Control and Rab40-TKO MDA-MB-231 cells expressing GFP-Rap1b were stained with Acti-stain 555 (phalloidin) and imaged with immunofluorescence microscopy. Quantification of GFP-Rap1b membrane localization **(J).**

## Description

Cell migration is a vital cellular process involving the structural polarization of the actin cytoskeleton to form distinct lagging and leading edges (Neumann and Prekeris, 2023; Schaks et al., 2019; Trepat et al., 2012). Formation of distinct cytoskeleton structures that drive cell migration requires tight spatiotemporal regulation of several small monomeric GTPases. While the Rho family of GTPases is widely considered to be the master regulators of the actin cytoskeleton (Kraynov et al., 2000; Kurokawa et al., 2003; Kurokawa and Matsuda, 2005; Montell et al., 2012; Wang et al., 2010; Watanabe et al., 1999) the Ras family of GTPases also recently emerged as one prominent group of migration regulators. The Ras subfamily (HRAS, KRAS, NRAS) are known for having activating mutations which drive cancer metastasis (Bos et al., 2007; Collins et al., 2023; Fuentes-Calvo et al., 2013). Less studied among the Ras family is the subfamily of Rap GTPases (Rap1a, Rap1b, Rap2a, Rap2b, Rap2c) which has also been implicated in regulating cell migration. Rap1 has been shown to regulate integrin trafficking during migration (Reedquist et al., 2000; Rothenberg et al., 2023), while we have shown that Rap2 regulates polarity and migration by inhibiting RhoA in lamellipodia ruffles (Neumann et al., 2025).

Recently, we have shown that Rap2 can be mono-ubiquitylated at three lysine residues (K117, K148, and K150) by a Rab40/Culllin5 (CRL5) E3 ubiquitin ligase complex located at the lamellipodia membrane (Duncan et al., 2022; Linklater et al., 2021). Mono-ubiquitination facilitates Rap2 GEF-dependent activation. Active Rap2 is then retained at the plasma membrane where it regulates lamellipodia dynamics (Duncan et al., 2022; Neumann et al., 2025). Here we sought to investigate if ubiquitylation by the Rab40/CRL5 complex is a conserved mechanism across the Rap subfamily of GTPases. Alignment of Rap1 and Rap2 shows strong conservation between Rap1 and Rap2. Notably, K117, K148, and K150 of Rap2 are all conserved in Rap1 (K117, K147, K149) indicating its possibility of being a Rab40/CRL5 complex substrate ( Fig. 1A ).

Next, we wanted to assess if Rap1 can bind to Rab40b as has been previously shown of Rap2 (Duncan et al., 2022; Duncan et al., 2021). To that end, recombinant purified GST-Rap1b beads were incubated with lysates generated from MDA-MB-231 cells stably overexpressing FLAG-Rab40b. Precipitate was analyzed by western blotting, using an anti-FLAG antibody to assess FLAG-Rab40b binding to GST-Rap1b. As shown in figure 1B-C, FLAG-Rap1b can bind to Rab40b with a slight preference for Rab40b in its GTP-bound state. To further examine the interaction between Rab40b and Rap1, we used a Rab40b-4A mutant which disrupts Rab40 binding to Cullin5 but preserves its ability to interact with its substrates. The Rab40b-4A mutant is unable to ubiquitylate and release its substrates, leading to an accumulation of Rab40-4A-ubiquitylation-substrate complexes as compared to Rab40-WT-ubiquitylation-substrate complexes (Duncan et al., 2022; Duncan et al., 2021). Consistent with the hypothesis that Rap1 is a Rab40/CRL5 substrate, Rab40b-4A mutation enhances Rap1b binding Rab40b, specifically when Rab40b is in its GTP-bound state ( Fig. 1D- E).

Our binding data suggest that Rap1 may be a substrate for the Rab40/CRL5 complex. To directly test this hypothesis, we next performed ubiquitylation assays using HEK293T cells overexpressing various combinations of constructs: 1) FLAG-Rap1b 2) FLAG-Rap1b + MYC-Ub 3) FLAG-Rap1b + MYC-Ub + HA-Rab40b 4) FLAG-Rap2a 5) FLAG-Rap2a + MYC-Ub 6) FLAG-Rap2a + MYC-Ub + HA-Rab40b. FLAG-Rap1b was then immunoprecipitated with an anti-FLAG antibody and blotted for ubiquitylation using an anti-MYC antibody. As shown in figure 1F, co-transfection of FLAG-Rap1b and MYC-Ub with HA-Rab40b increases MYC-ub signal upon FLAG-Rap1b immunoprecipitation. Furthermore, the bands observed with the anti-MYC antibody show that the Rap1b ubiquitylation pattern is the same as the pattern observed for FLAG-Rap2a ( Fig. 1F ). We previously demonstrated that Rab40/CRL5 mediates mono-ubiquitylation of Rap2a at K117, K148, and K150 ( Fig. 1F, (Duncan et al., 2022)). Thus, this experiment suggests that Rap1b is also a substrate of the Rab40/CRL5 complex and is capable of being mono-ubiquitylated at the conserved lysine residues K117, K147, and K149.

The similarity between Rap1 and Rap2 in being substrates of the Rab40/CRL5 complex suggests that Rap1 ubiquitylation may also be necessary for Rap1 activation. We tested this hypothesis using GST-RalGDS pulldown assays. RalGDS is a well-known effector of Ras and RAP GTPases. Interaction of Rap with the Ras-binding-domain of RalGDS is commonly used to measure Rap activity (Duncan et al., 2022; Spaargaren and Bischoff, 1994). As such, we incubated GST-RalGDS beads in lysates from either control cells or Rab40-TKO (Rab40a, Rab40b, Rab40c co-knock-out) MDA-MB-231 cells overexpressing GFP-Rap1b. Our data show that GFP-Rap1b binds better to GST-RalGDS in control cells as compared to Rab40-TKO cells ( Fig. 1G- H), indicating that Rab40-dependent ubiquitylation is necessary for Rap1b activation.

Rab40/CRL5 regulation of Rap1b activity suggests that it may also regulate Rap1b localization. Similarly to Rap2, Rap1 has been shown to be localized to the plasma membrane where it was proposed to regulate cell-cell adhesions (Rothenberg et al., 2023). However, it is also known to localize to the nuclear envelope (Liu et al., 2010). We have shown that mono-ubiquitylation of Rap2 drives its localization to the lamellipodia and plasma membrane while loss of ubiquitylation results in Rap2 internalization and trafficking to lysosomes (Duncan et al., 2022). We predicted that, like Rap2, loss of ubiquitylation would drive Rap1b away from the plasma membrane and nuclear membrane, resulting in its lysosomal accumulation. We tested this prediction by using immunofluorescence microscopy to visualize GFP-Rap1b localization in control and Rab40-TKO MDA-MB-231 cell lines. In control cells, GFP-Rap1b displays strong localization to the nuclear envelope while showing weaker, yet distinct localization to regions of plasma membrane ruffling. Unexpectedly, loss of Rab40 reduced GFP-Rap1b localization from the nuclear envelope and cytoplasm and drastically increased its localization to ruffling membrane edges ( Fig. 1I- J). Thus, while we show that both Rap1 and Rap2 are regulated and activated by Rab40/CRL5-dependent mono-ubiquitylation, the spatial localization of mono-ubiquitylation dependent regulation is different for Rap1 and Rap2. Additional studies will be needed to fully understand the role of mono-ubiquitylation in regulating Rap1 function.

Taken together, our study suggests a conserved mechanism of mono-ubiquitylation regulating the Rap subfamily of GTPases. However, while mono-ubiquitylation is necessary for activation of both Rap1 and Rap2, mono-ubiquitylation dependent localization is different between Rap1 and Rap2. The Rab40/CRL5 complex is localized to the lamellipodia membrane (Duncan et al., 2022; Duncan et al., 2021). Thus, mono-ubiquitylation may present itself as a mechanism by which lamellipodia localized Rap GTPases can be spatially segregated. In accordance with this idea, Rap1 and Rap2 have been reported to have antagonistic roles in regulating endothelial barrier stiffness (Pannekoek et al., 2013). If Rap1 and Rap2 play antagonistic roles in lamellipodia ruffling, mono-ubiquitination could be the mechanism spatiotemporally regulate their localization, allowing for both Rap1 and Rap2 function. Interestingly, Ras GTPase family member K-Ras has also been shown to be mono-ubiquitinated at K117 which also promotes its activity (Choi et al., 2018; Osaka et al., 2021; Sasaki et al., 2011). Thus, mono-ubiquitination may present itself as a unique mechanism regulating all Ras family GTPases, making it an important modification for further study in the context of cell migration.

## Methods

**Cell culture**

MDA-MB-231 cells were cultured in 231 media (DMEM with 4.5 g/liter glucose, 5.84 g/liter L-glutamine, 1% sodium pyruvate, 1% nonessential amino acids, 1 µg/ml insulin, 1% penicillin/streptomycin, and 10% FBS). HEK293T cells were cultured in 293T media (DMEM with 4.5 g/liter glucose, 5.84 g/liter L-glutamine, 1% penicillin/streptomycin, and 10% FBS). The Rab40-TKO line had been previously created and validated (Linklater et al., 2021). Cell lines were routinely tested for mycoplasma. Additionally, all cell lines were authenticated in accordance with ATCC standards.

**Generation of lentiviral stable cell lines**

Calcium Phosphatase was used to transfect HEK293T cells (50% confluent) with pLVX plasmid containing GFP-tagged gene of interest. After 6 hours the media was replaced with fresh 293T media. Cells were left for 48 hours to allow for the virus to accumulate in the media. Viral 293T media was collected, filtered through a 0.45 µm PVDF low binding syringe filter, and treated with polybrene (100 µg per 1 mL media). Viral 293T media was added to target MDA-MB-231 cells (50% confluent) for 2 hours. Media was replaced with 231 media and target cells were allowed to recover for 24 hours, then selected with puromycin (5 µg/mL). Expression for the GFP-tagged gene of interest in cell lines were then validated using Western Blotting.

**Rab40 and Rap1 binding assays**

Cell overexpressing FLAG-Rab40b-WT or FLAG-Rab40b-4A were lysed in buffer containing 20 mM Hepes, pH 7.4, 150 mM NaCl, 1% Triton X-100, 1 mM PMSF, and 5 mM iodoacetamide (DUB inhibitor) on ice for 30 mins. Lysate was clarified in chilled microcentrifuge at 21,000xg. 25 µg of lysate was taken as an input control. Lysates were then distributed equally to have 400 µL at 3 mg/mL. lysates were locked with GTP or GDP using the following steps. 1) 5mM EDTA was added to lysate for 5 mins. 2) 5mM GppCp or GDP was added to lysate for 5 mins. 3) 15 mM MgCl _2_ was added to lysate from a stock of MgCl _2_ diluted to 150 mM in water, pH 7.0 for 5 mins. 10 µg of GST or GST-Rap1b (purified as previously describe for GST-Rap2a, (Duncan et al., 2022)) was added to the lysate and the mixture was rotated at room temperature for 1 hour. 75 µL of GST beads were then added to the tubes and the mixture and rotation was continued for 30 minutes. Beads were washed 5x with 1 mL of buffer containing 20 mM Hepes, pH 7.4, 300 mM NaCl, and 0.1% Triton X-100. Protein was eluted from beads in 35 µL of 1x SDS sample loading dye, separated by SDS-PAGE and analyzed by Coomassie staining and western blot. 20 µL of elution and lysate controls were used for western blotting. 10 µL of elution was used for Coomassie staining to confirm the presence of GST/GST-RalGDS.

**Rap1/2 Ubiquitylation Assay**

Briefly, HEK293T cells (∼80% confluency) were transfected with plasmids expressing pRK5-FLAG-Rap2a or FLAG-Rap1b with or without pRK5-Myc-Ub, pRK7-HA-Rab40b using Lipofectamine 2000. After 24 h, cells were harvested and lysed in 1 ml lysis buffer (20 mM Tris-HCl, pH 7.6, 150 mM NaCl, 2 mM EDTA, 1% Triton X-100, and 10% glycerol) with 1% SDS. Cell lysates were collected and boiled at 95°C for 10 min. Supernatants were then diluted with lysis buffer to reduce SDS concentration to 0.1% and incubated with 5 µg anti-FLAG M2 antibody and 60 μl 50% protein G bead slurry overnight. Beads were pelleted by centrifugation and washed three times with lysis buffer plus 0.5 M NaCl. Bound proteins were eluted in 50 μl of 1× SDS sample buffer. Eluates (20 μl) were resolved via SDS-PAGE and transferred to nitrocellulose membranes for immunoblotting. To remove the background IgG heavy chain and light chain after immunoprecipitation, we used an IgG light chain–specific secondary antibody and FLAG antibody directly conjugated with HRP. Blot images were captured using a ChemiDoc MP Imaging system.

**Active Rap1 pulldown assays**

GST-RalGDS was purified as previously described (Duncan et al., 2022). MDA-MB-231 cells stably expressing GFP-Rap were grown in 10cm plates to 90% confluency, then harvested and pelleted (frozen if necessary). Pellets were lysed in buffer containing 20 mM Hepes, pH 7.4, 150 mM NaCl, 1% Triton X-100, 1 mM PMSF, and 5 mM iodoacetamide (DUB inhibitor) on ice for 30 mins. Lysate was clarified in chilled microcentrifuge at 21,000xg. Lysate was brought to equal concentration and volume in buffer containing 20mM Hepes pH 7.4 and 150mM NaCl. 20 µg of either GST (control) or GST-RalGDS was added to the lysate. Lysate controls (equal µg across conditions) were also taken for later use. Tubes with the GST proteins and lysate mixture were rotated for 60 mins at room temperature. 45 µL of glutathione beads (50% in PBS) were added to the tubes, and rotation was continued for 30 more minutes. Beads were then washed 5x in 1 mL buffer containing 20 mM Hepes, pH 7.4, 300 mM NaCl, and 0.1% Triton X-100. Protein was eluted from beads in 35 µL of 1x SDS sample loading dye, separated by SDS-PAGE and analyzed by Coomassie staining and western blot. 20 µL of elution and lysate controls were used for western blotting. 10 µL of elution was used for Coomassie staining to confirm the presence of GST/GST-RalGDS.

**Immunofluorescence staining**

MDA-MB- 231 cells were seeded onto 1X collagen-coated glass cover glass slips and grown in full growth media for approximately 24 hours. Cells were later washed with room temperature and fixed with 4% Paraformaldehyde for 15 minutes at RT. Cells were then quenched for 5 minutes with Quench buffer (375 mg of glycine diluted in PBS), and incubated in Incubation buffer (1ml of FBS, PBS, 1% BSA, 200mg saponin) for 30 minutes. Acti-stain 555 (phalloidin) was reconstituted in methanol and diluted at 1:100 in incubation buffer. Diluted Acti-stain 555 was incubated with cells for 30 minutes in at humidified chamber. Cells were treated with 1:1600-1:2000 Hoechst stain for 5 minutes, then washed twice with PBS. Coverslips were then mounted onto glass slides using Vectashield. The intracellular/Whole cell fluorescence ratio of GTP-Rap1b was calculated in Fiji using images that were max projected on the stacks where GFP-Rap1b signal was in focus. Per cell, the actin channel was used to define the cell perimeter. An ROI was traced on the outside of the actin channel and defined as the whole cell while another ROI was traced on the inside of the actin channel and defined as the intracellular ROI. The ROIs were then applied to the GFP-Rap2a channel and the integrated density was measured. ROIs were also used to measure the integrated density of the background fluorescence. Data was expressed as a ratio of integrated density which was defined as

$$\frac{\mathrm{intracellular}-\mathrm{background}}{\mathrm{whole}-\mathrm{background}}$$

where a value of 1 would indicate all the signal was intracellular.

**Image acquisition**

All fixed imaging was performed on a widefield inverted Zeiss Axiovert 200M microscope using a 63X oil-objective, QE charge-coupled device camera (Sensicam), and Slidebook v. 6.0 software (Intelligent Imaging Innovations). Images were taken as z-stacks with 0.5 µm step intervals. Images were deconvolved (Nearest Neighbors) using the Intelligent Imaging Innovations software. Further image processing was performed in Fiji software.

**Statistical analysis**

Graphs are displayed as biological replicates (opaque dots) overlayed on technical replicates (translucent dots). Error bars represent the standard deviation of the biological replicates. Replicates are displayed by color. Statistical analysis was performed on biological replicates which were calculated from the mean of the technical replicates. For all analyses comparing 3 or more conditions, a one-way ANOVA was used with comparisons run for each column against the mean of each other column. p-values of interest are displayed on graphs. For all analyses comparing 2 conditions, a student’s t-test was used with all significant p-values displayed.

## Reagents

**Table:** 

**Name**	**Company/Product Number**	**Dilution**
FLAG M2	Sigma, F3165, mouse	1:1000
GFP	Proteintech, 1E10H7, mouse	1:1000
FLAG-HRP conjugate	CST-86861, rabbit	1:1000
light chain–specific secondary antibody	Jackson; 211-032-171	1:1000
IRDye 680RD Anti-Mouse Secondary	Li-Cor 926-68072	1:5000
IRDye 800CW Anti-Rabbit Secondary	Li-Cor 926-32213	1:5000
Acti-stain 555 (phalloidin)	Cystoskeleton, PHDH1-A	1:100
Protein G-Sepharose	Cytiva 17-0618-01	50%
Glutathione Agarose Resin	GoldBio, G-250	50%
GppCp (non-hydrolyzable GTP analog)	Abcam, ab146660	N/A
Hoechst 33342	AnaSpec AS-83218	1:4000
Lipofectamine 2000	Invitrogen 11668027	N/A
Bio-Rad Protein Assay Dye Reagent (Bradford method)	Bio-Rad 5000006	1:5
Intercept TBS Blocking Buffer	Li-Cor 927-60001	1:3
